# Assessment of a conservative approach for restoration of extensively destroyed posterior teeth

**DOI:** 10.1590/1678-7757-2018-0631

**Published:** 2019-07-25

**Authors:** José MONDELLI, Fabio Antonio Piola RIZZANTE, Fabiano Bassalobre VALERA, Renato ROPERTO, Rafael Francisco Lia MONDELLI, Adilson Yoshio FURUSE

**Affiliations:** 1 Marília São Paulo Brasil Private practice, Marília, São Paulo, Brasil.; 2 Case Western Reserve University Case Western Reserve University School of Dental Medicine Cleveland Ohio United States of America Case Western Reserve University, School of Dental Medicine, Cleveland, Ohio, United States of America.; 3 Universidade de São Paulo Universidade de São Paulo Faculdade de Odontologia de Bauru Departamento de Dentística, Endodontia e Materiais Odontológicos Bauru São Paulo Brasil Universidade de São Paulo, Faculdade de Odontologia de Bauru, Departamento de Dentística, Endodontia e Materiais Odontológicos, Bauru, São Paulo, Brasil.

**Keywords:** Composite resins, Dental cavity preparation, Endodontically treated teeth, Permanent dental restorations

## Abstract

Extensive restorations in posterior teeth always bring doubts to the clinicians regarding the best protocol, mainly when structures of reinforcement were lost.

**Objective:**

This study aimed to evaluate the effect of beveling on the fracture resistance and pattern of class II (MOD) restored teeth.

**Methodology:**

Ninety human premolars were randomly assigned into 9 groups: CTR (control/sound); NC (cavity preparation, non-restored); RU (restored, unbeveled); RTB (restored, entire angle beveling); RPB (restored, partial/occlusal beveling); EC (endodontic access/EA, non-restored); EU (EA, unbeveled); ETB (EA, entire angle beveling); EPB (EA, partial/occlusal beveling). Teeth were restored with Esthet X resin composite and stored in distilled water for 24 h before the inclusion in PVC cylinders. The axial loading tests were performed with 500 kgF at 0.5 mm/min crosshead speed until fracture of the specimens. Fracture resistance and pattern were accessed and data were analyzed using one-way ANOVA and Tukey’s HSD test (α=0.05).

**Results:**

Mean (±SD) failure loads ranged from 136.56 (11.62) to 174.04 (43.5) kgF in the groups tested without endodontic access. For endodontically accessed teeth, fracture resistance ranged from 95.54 (13.05) to 126.51 (19.88) kgF. Beveling of the cavosurface angle promoted the highest fracture resistance values (p<0.05) and prevented catastrophic fractures.

**Conclusions:**

Cavosurface angle beveling is capable of improving fracture resistance and pattern for both endodonticaly accessed and non-accessed teeth.

## Introduction

Restoration of extensively destroyed tooth aims to reestablish function and aesthetics. Extensive cavities are directly associated with lower fracture resistance^1 - 3^ and are often associated with marginal failures, cracks and total/partial cusp fracture.^4^ This seems to be true for posterior teeth, especially for upper premolars, in which coronary anatomy tends to deflection and separation of the cusps during mastication.^2 , 5 - 7^ The depth of the cavity and the involvement of reinforcement structures such as marginal crests and pulp chamber roof (endodontic access) further increase tooth structure deflection as well as stress concentration at the buccal pulpal and lingual pulpal angles.^3 , 4 , 8 - 10^

Despite the large indications for cusp coverage (i.e. crown, onlay, etc) in restorations of extensively compromised teeth,^8 , 11^ resin composite restorations are cheaper and have adequate physical and mechanical properties such as adhesiveness, elasticity, resilience, and resistance to tensile, shear and compression stresses, thus allowing greater synergy between tooth and restorative material, capable of absorption of masticatory forces^2 - 6 , 12^ and reduction in cusp deflection through cusp “splinting”.^4 - 7 , 13 , 14^ Moreover, the adhesive concepts allow more conservative cavity designs, improving the resistance of tooth reminiscent.^1 - 3^

Bonding in enamel presents long-term stability. However, enamel is composed of prisms that are often perpendicular to the enamel-dentin junction and can be fractured if the forces are not parallel to this direction.^15 , 16^ Micro-cracks and consequent degradation of enamel can be prevented by beveling the cavosurface angle and using adequate polymerization and polishing techniques etc. Beveling provides a smoother and more regular enamel surface through the removal of weakened prisms, which could fracture due to polymerization stress of composites, as well as increase the surface area to bonding, contributing to a more stable restoration.^15 - 22^ Even with all these advantages reported and proved, some clinicians often relegated and even contraindicate beveling claiming it does not promote better restoration performance and/or promotes a lower thickness of the restorative material, over extension of cavity margins and exposure of the restorative material/tooth interface at areas of occlusal contact.^23 - 25^

This article aimed to evaluate the effect of bevel on the fracture resistance and pattern in MOD class II cavity preparations, with and without endodontic access, after restoration with direct composite. The null hypothesis tested was that there should be no difference between the different cavosurface angle treatments considering the resistance and fracture pattern of direct composite restored teeth, with or without endodontic access.

## Methodology

This investigation had dental preparation (in nine levels) as study factor: positive control (without preparation), two negative control groups (preparations with and without endodontic access and without restoration), and six groups according to enamel beveling and presence of endodontic access (no-enamel bevel, occlusal enamel bevel or entire cavosurface angle beveling, with and without endodontic access). The response variables were fracture resistance (evaluated using a universal testing machine) and fracture pattern (evaluated on a stereomicroscope).

**Figure 1:**
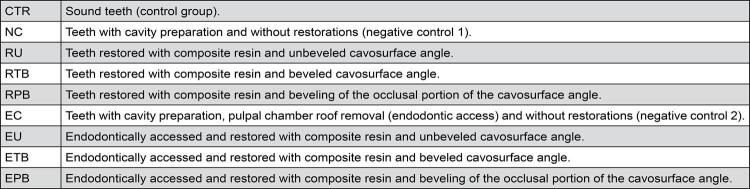
Group division according to the different treatments

After approval of the local Research Ethics Committee, ninety sound human maxillary premolars with similar dimensions and without any cracks or malformations, extracted due to orthodontic or periodontal reasons, were selected and randomly assigned to 9 different groups (n=10) ( Figure 1 ).

Standardized cavity preparations were performed by a single operator using high-speed handpiece, under water cooling and with brand-new burs (up to 4 preparations before recycling). All cavity measures were double-checked with periodontal probe and digital caliper. Cavities were made auto retentive by using 245 carbide burs (KG Sorensen, Cotia, SP, Brazil).

### Cavity characteristics:

- Pulpal wall: flat and perpendicular to longitudinal axis of the tooth with 2 mm in depth and 1/3 of isthmus aperture;
- Buccal and lingual/palatal walls: flat and convergent to occlusal with 2 mm in depth;
- Gingival wall: flat and parallel to the pulpal wall with 1.5 mm thickness and 1/3 of isthmus aperture. All cervical margins were determined in the enamel.
- Axial walls: Flat, convergent to occlusal with 1.5 mm in depth.

Considering the groups with endodontic access, in addition to the previous characteristics, the pulpal chamber roof was removed (with n.4 round bur – KG Sorensen), to simulate an endodontic access. Consequently, there were no axial walls.

For the groups RTB, RPB, ETB, and EPB, a concave bevel was made with a flame-shaped diamond bur (#1111, KG Sorensen) in slow speed and with approximately 0.5 mm in length. The length of the bevel was checked with a digital caliper.

After cavity preparation, prophylaxis with pumice was performed on the specimens followed by restoration with a direct adhesive restoration system (Esthet X – Dentsply, York, PA, USA).

For teeth with endodontic access, the pulp chamber was filled using a resin-modified glass ionomer cement (Vitremer, 3M ESPE, Saint Paul, MN, USA), following the manufacturer’s recommendations: primer application for 30 s, solvent evaporation with air and light curing for 20 s using a 540 mW/cm^2^ light unit (XL 3000, 3M ESPE, Saint Paul, MN, USA); followed by manipulation of Vitremer 1 powder: 1 liquid, insertion in the pulp chamber using a syringe system (until the material was flat with the proximal boxes of the class II cavity), and light curing for 40 s.

All enamel and dentin walls (including the bevel) were etched with a 35% phosphoric acid gel (Dentsply) – 30 s for enamel and 15 s for dentin. Specimens were washed for 30 s and dried with absorbing paper followed by application of 2 layers of bonding agent (Prime&Bond NT – Dentsply). A 5 s gentle air blast was applied to evaporate the adhesive solvent followed by 10 s light curing.

The restorative materials were inserted following the oblique incremental technique intercalated with 40 s light curing, starting with the proximal boxes. After restoration, 40 s additional light curing was performed for each surface (mesial, distal and occlusal). Gross excess materials were removed with #12 scalpel blade.

Samples were stored in distilled water at ambient temperature (23±2°C) for 24 h. Restoration finishing and polishing was performed with # 3118/1190 F and FF diamond burs (KG Sorensen) and yellow/white Viking silicon abrasive tips (KG Sorensen) associated with resin composite lubricating gel. Then, teeth were included in 20 mm diameter and 30 mm height self-curing polystyrene resin (100 mL resin: 2 mL catalyst) using PVC cylinders until 2 mm below the cementoenamel junction, simulating the position of the alveolar bone crest. A metallic bar was used to ensure the positioning of the cusps parallel to the acrylic base, and the specimens were stored for 7 day in distilled water at ambient temperature.

Axial loading tests were performed using a universal testing machine (EMIC DL 2000, EMIC, São José dos Pinhais, PR, Brazil), with 500 KgF loading cell and 0.5 mm/min crosshead speed, until fracture of the specimens. The compression test was performed with an 8 mm diameter steel cylinder, adapted in a metallic bar with 13 cm in length perpendicular to the intercuspal axis. Internal portion of cusps were prepared with 8 mm cylindrical aluminum oxide burs to ensure maximum adaptation with the steel cylinder and to prevent it from sliding and/or incorrect force direction. For each specimen, the result of fracture resistance and fracture pattern were recorded after the axial compression test.

**Figure 2:**
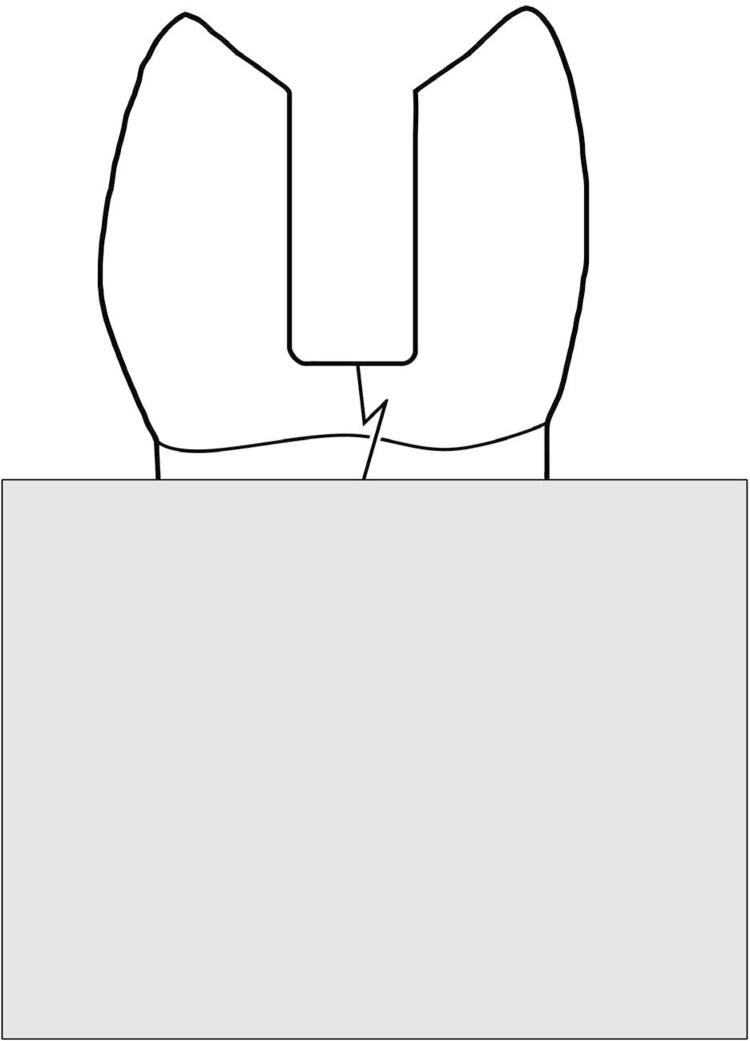
Schematic drawing representing a longitudinal fracture

**Figure 3:**
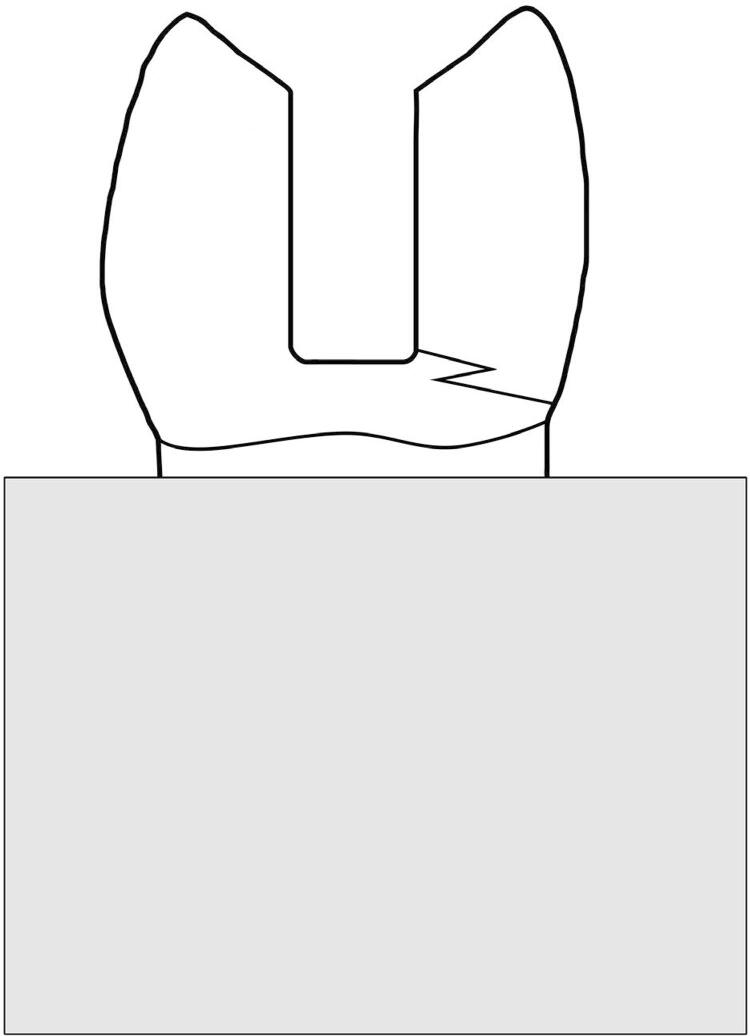
Schematic drawing representing an oblique fracture

Considering the fracture pattern, the specimens were classified in 2 groups: oblique (1) and longitudinal (2). The results were analyzed by Shapiro-Wilk normality test, one-way ANOVA and Tukey Kramer tests, all with p<0.05. In addition, a descriptive analysis was used to calculate the relative percentage of the fracture patterns observed for each group. Figures 2 and 3 show drawings of the fracture patterns observed.

## Results

### Teeth without endodontic access/treatment

Results showed statistically significant difference considering fracture resistance, except for the groups RU/RPB and RTB/RPB, which were similar to each other. Group RTB showed the highest fracture resistance, corresponding to approximately 85% of the sound tooth resistance. The fracture pattern for the negative control group couldn’t be classified because of its variability. For the other groups, the inversion of the fracture pattern could be observed from groups without bevel (RU) for total cavosurface angle beveling (RTB) ( Figure 1 ).

### Teeth with endodontic treatment/access

Results showed statistically significant difference considering fracture resistance. Group ETB showed the highest values for fracture resistance and was statistically similar to group EPB, but still representing about 50% of the resistance of a sound tooth. Considering fracture pattern, occlusal beveling (group EPB) was not capable of changing the fracture pattern when compared with the EU (without beveling), while the beveling of the entire cavosurface angle (group ETB) was able to prevent catastrophic fractures ( Table 1 ).

**Table 1:** Mean values (standard deviation) of fracture resistance and fracture pattern of teeth without and with endodontic access

Group	Endodontic access	Fracture Resistance (KgF)	Fracture pattern (oblique or long axis)
CTR	-	252.41(24.08)^A^	50% oblique
NC	No	95.6 (26.66)^B^	Much variable
RU	No	136.53 (11.62)^C^	80% long axis
RTB	No	213.55 (11.20)^D^	100% oblique
RPB	No	174.04 (43.5)^CD^	90% oblique
EC	Yes	77.59 (16.43)^B^	Much variable
EU	Yes	95.54 (13.05)^B^	70% long axis
ETB	Yes	126.51 (19.88)^C^	90% oblique
EPB	Yes	100 (30.17)^BC^	60% long axis

Different superscript letters indicate statistically significant differences (p<0.05)

## Discussion

Null hypothesis was rejected because different cavosurface angle treatments influenced fracture resistance and fracture pattern of the groups tested. Cavity preparations in teeth have shown problems related to the fracture resistance of the reminiscent,^1 , 9 , 10 , 12 , 13 , 26 , 27^ and can be responsible for opening of restoration margins, cusp cracks and fractures. Reduction in fracture resistance is even higher considering teeth with endodontic access, and this is related to the removal of reinforcement structures (marginal crests and pulpal chamber roof).^2 - 4 , 6 , 11^ This is in agreement with the present study as the negative control groups (NC and EC) showed statistically lower fracture resistance when compared with the sound teeth group (CTR). All groups with endodontic access showed lower values when compared with the groups without endodontic access. These results confirmed that tooth structure removal can be correlated with lower fracture resistance values, and up to 90% reduction can be observed in premolars.^2 , 13 , 27 , 28^ This high reduction in resistance can be associated with an “enlargement” of cusps due to premolar teeth anatomy, which may cause a tendency for separation of cusps during masticatory efforts because the restoration acts like a wedge between buccal and lingual cusps, which could result in catastrophic fractures.^4 , 5 , 13 , 28 - 30^

An operative maneuver for such situations consists of cusp reduction and covering with indirect restorations to protect the reminiscent tooth structure.^4 , 7 , 8 , 30^ Since adhesive restorations can achieve good results in the major part of the cases, reestablishing part of the tooth resistance, such restorations can also be performed with cusp reduction or splinting with resin composites.^2 , 6 , 7 , 13 , 14 , 28 , 30 , 31^ These results are in agreement with the present study, in which the entire beveling of the cavosurface angle was capable of improving the fracture resistance of the restored teeth simulating the effect of an onlay restoration ( Table 1 ).

However, removal of reinforcement structures (i.e. marginal crests), including pulpal chamber’s roof removal, has direct impact on the restored tooth resistance^1 , 2 , 6 , 8 , 32^ . Considering endodontically accessed teeth, only groups with the entire cavosurface angle beveling showed fracture resistance increase when compared with the negative control (NC) ( Table 1 ). This can be explained by the increase in bonded surface area and better enamel prisms orientation (transversal), resulting in better adhesion and marginal adaption, as well as better force distribution.^7 , 15 - 17 , 33 - 35^ Such explanations are in accordance with this article results, in which fracture resistance values were increased in the groups with cavosurface angle beveling. The reestablishment of fracture resistance of dental reminiscent is important as well as the fracture pattern that can determine the maintenance of the tooth inside the oral cavity if the restoration fails. Longitudinal fractures usually divide the teeth into two parts, and their extraction is recommended. Oblique fractures are usually restorable.

One can notice an inversion in the fracture pattern from the groups without beveling (groups RU and EU) to the groups with cavosurface angle beveling (groups RTB and ETB). That said, direct composite by itself was unable to reestablish fracture patterns similar to those from sound teeth, but it was capable of restoring the fracture pattern protecting the teeth against catastrophic failure in association with cavosurface angle beveling. The protection provided by the entire cavosurface angle beveling was similar to that reported for onlays,^8 , 13 , 27^ despite the lower fracture resistance values when compared with sound teeth.

The results shown in this study considering fracture resistance and fracture pattern reinforces the use of the cavosurface angle beveling to improve the resin composite restoration performance and reliability ( Table 1 ). This suggests that increase in bonded area and surface quality allows a better force distribution through the teeth reminiscent.

In the best of authors’ knowledge, no studies assessed beveling as a conservative approach for cusp splinting, preventing direct comparison. Nevertheless, this study showed promising results, similar to the above discussed studies assessing fracture resistance with onlays and/or direct restorations with cusp coverage. Future studies should be performed to assess the fracture resistance and pattern under angular loadings, since occlusal forces might dissipate through the restorative material. The use of premolars for such tests are ideal due to their anatomy, resulting in enlarged cusps after removal of the roof of the pulp chamber and marginal crests in MOD class II cavity preparations. This fact, associated with axial loading forces without simulation of periodontal ligament (avoiding any “cushion effect”), corresponds to the worst case scenario during tests with axial loading.^4 , 5 , 13 , 14^

Regarding the method, some issues should be addressed. A two-step etch-and-rinse adhesive was used. Although differences in bond strength values are expected considering different bonding approaches (i.e. three- and two-step etch-and-rinse, two- and one-step self-etching and universal), the overall risk ratio of postoperative sensitivity for resin composite restorations placed in cervical lesions and the clinical service are not different when either etch-and-rinse or self-etching adhesives are used.^36^ Moreover, the bonding of two-step etch-and-rinse adhesives to enamel is not affected by the small differences in the application protocol such as in moisture.^37^ The fracture resistance was evaluated after a 7-day storage in distilled water and no thermocycling or further aging was conducted. This was done since this study focused on assessing the influence of enamel beveling on the fracture resistance and pattern in class II restorations with and without endodontic access to provide more comprehension about the beveling *per se* . Including artificial aging methods in this protocol, addressing the long-term decrease in the bond strength of the resin composite would be interesting, but it would add more variables, and the results could be far more complex to be explained.

In summary, the removal of tooth structure decreases fracture resistance, and restorations are not capable of fully restoring tooth resistance. Nevertheless, beveling of the entire cavosurface angle in class II MOD restorations can improve fracture resistance and fracture pattern of teeth restored with direct composite, and might be recommended in such situations.
